# Effectiveness of intraoperative blood recovery in surgical patients at risk of transfusion: A rapid review

**DOI:** 10.1016/j.htct.2026.106517

**Published:** 2026-09-07

**Authors:** Liza Yurie Teruya Uchimura, Tatiana Yonekura, Jeane Roza Quintans, Mabel Fernandes Figueiró, Priscila Murador, Marcelo Addas de Carvalho

**Affiliations:** a Hcor, Sao Paulo, Brazil; b Coordenação-Geral de Sangue e Hemoderivados (CGSH), Departamento de Atenção Especializada e Temática (DAET), Secretaria de Atenção Especializada à Saúde (SAES), Ministério da Saúde do Brazil, Brazil; c Centro de Hematologia e Hemoterapia (Hemocentro-UNICAMP), Universidade Estadual de Campinas (UNICAMP), Campinas, Brazil

**Keywords:** Blood transfusion, Autologous, Operative blood salvage, Cell saver

## Abstract

**Background:**

Allogeneic blood transfusion is widely used to manage surgical blood loss but carries risks, such as infections and transfusion reactions, and is affected by blood shortages. Intraoperative blood recovery (cell salvage) may reduce the need for donor blood, improving patient safety and resource availability. This rapid review evaluated its clinical effectiveness, safety, and costs in surgical patients at risk of transfusion.

**Methods:**

Following the Cochrane rapid review guidelines, PubMed, the Virtual Health Library, and the Cochrane Library were searched for systematic reviews, randomized and non-randomized trials, and observational studies published between 2004 and October 2024. Eligible studies compared intraoperative blood recovery with allogeneic transfusion or no intervention in surgical patients. Outcomes included transfusion requirements, clinical safety, hospital indicators, and costs. Study quality was assessed using the AMSTAR-2 and Joanna Briggs Institute tools.

**Results:**

Fifty-two studies across multiple surgical specialties were included. Intraoperative blood recovery consistently reduced allogeneic transfusion requirements in orthopedic, obstetric, gastrointestinal, and pediatric cardiac surgeries. Evidence was mixed for cardiac, thoracic, and vascular surgeries. Safety profiles were favorable, with no significant adverse events directly attributed to intraoperative blood recovery. Some studies reported reductions in infection rates and hospital stay. Cost analyses (n = 16) yielded mixed results, with eight studies showing cost savings due to reduced transfusion needs.

**Conclusion:**

Intraoperative blood recovery is effective and safe in reducing allogeneic transfusions, especially in high-blood-loss surgeries. Cost-effectiveness is context-dependent. Implementation should prioritize high-risk procedures and align with patient blood management strategies.

## Introduction

Allogeneic blood transfusion remains a primary and often indispensable strategy for managing intraoperative blood loss, despite its well-documented risks, such as transfusion reactions, infections, and prolonged hospital stays [1,2]. Intraoperative blood recovery or cell salvage has gained attention not only for its potential to reduce reliance on donor blood and minimize transfusion-related complications, but also addressing broader systemic challenges, including limited blood availability and inequitable access to safe blood products [1].

Allogeneic blood transfusion faces significant challenges, including donor shortages and ethical concerns, such as refusal of blood products for religious reasons. Despite numerous initiatives and interventions to improve the availability and safety of blood products, blood transfusion management remains a major public health concern, often due to insufficient donor supply and limited access to specialized equipment and services [2].

While previous studies have highlighted positive trends, such as the need for fewer transfusions and improved patient safety, the effectiveness of intraoperative blood recovery across different surgical subspecialties remains underexplored, particularly regarding the identification of optimal clinical indications. This rapid review was conducted to assess the clinical effectiveness, safety, and cost-effectiveness of intraoperative blood recovery in surgical patients at risk of allogeneic transfusion, with the aim of supporting evidence-informed decisions in health policy and clinical practice.

## Methods

### Study design

This rapid review was conducted in accordance with the methodological guidelines of the Cochrane Rapid Reviews Methods Group (RRMG) to ensure rigor and transparency throughout the evidence synthesis process [3]. Rapid reviews are a streamlined form of systematic review designed to deliver timely evidence by employing methodological shortcuts which expedite the processes of identifying, selecting, extracting, and synthesizing data to address urgent or emerging healthcare issues [4]. These strategies aim to accelerate the review process while minimizing the risk of bias and maintaining acceptable methodological quality [4].

The rapid review format was adopted not only for reasons of methodological feasibility, but also the need to provide timely evidence to inform policy decisions. Specifically, this review was commissioned to support the Brazilian Ministry of Health in its evaluation of the clinical effectiveness of intraoperative blood recovery in surgical patients at risk of requiring transfusion support, with the aim of guiding evidence-informed decisions regarding its potential implementation within the national healthcare system.

The protocol of this review was registered in advance on the Open Science Framework (OSF) platform (https://doi.org/10.17605/OSF.IO/4G9WY).

This review addressed the following research question: What is the effectiveness of intraoperative blood recovery in surgical patients at risk of requiring transfusion support? The question was developed using the PICOS framework. The population consisted of surgical patients considered at risk of needing transfusion support. The intervention under analysis was intraoperative blood recovery. The comparator was either allogeneic blood transfusion or no comparator. The primary outcomes of interest were the effectiveness of the intervention and the need for transfusion. Secondary outcomes included associated costs, and hospital indicators, such as length of hospitalization, infection rate, mortality, and readmission. The type of surgical procedure and patient profile were considered study characteristics. Eligible study designs included systematic reviews with or without meta-analysis, published from 2004 onwards, as well as randomized controlled trials, non-randomized clinical trials, and observational studies.

### Search strategy

A literature search was conducted on October 2, 2024 of the following electronic databases: PubMed, Virtual Health Library (VHL), and the Cochrane Library. Controlled vocabularies from each database were combined with relevant synonyms and keywords to formulate the search queries. The complete search strategies, with the included keywords, are detailed in Appendix 1.

### Study selection and eligibility criteria

The methodological shortcuts taken were to limit the review to studies published in Portuguese, English or Spanish. The title and abstract screening stage of the review process was conducted by pairs of reviewers working independently. Assessments of the eligibility, data extraction, and quality appraisal were performed independently by reviewers, and any discrepancies were resolved by consensus.

After duplicate records were removed using Mendeley software, the remaining citations were imported into the Rayyan platform for the title and abstract screening phase. To ensure consistency among reviewers in applying the inclusion and exclusion criteria, a calibration exercise was conducted using a sample of studies.

### Data extraction

Data were extracted independently by four reviewers using a standardized Excel spreadsheet. The data collected included the study reference (title, authors, publication year), objective, design, application context (such as emergency or elective surgery), characteristics of the intraoperative blood recovery method, patient characteristics, the surgical procedures where the method was applied, and the outcomes reported. These outcomes included the clinical effectiveness of the intervention, the total number and mean volume of transfusions in the intervention and control groups, associated costs, healthcare utilization metrics, and the reported benefits or limitations of the method.

### Quality assessment of included studies

The methodological quality of the included studies was assessed by one reviewer and checked by a second reviewer. The AMSTAR 2 tool was used for systematic reviews and the Joanna Briggs Institute critical appraisal tools were applied for all other study designs [5].

## Results

**Fig. 1:**
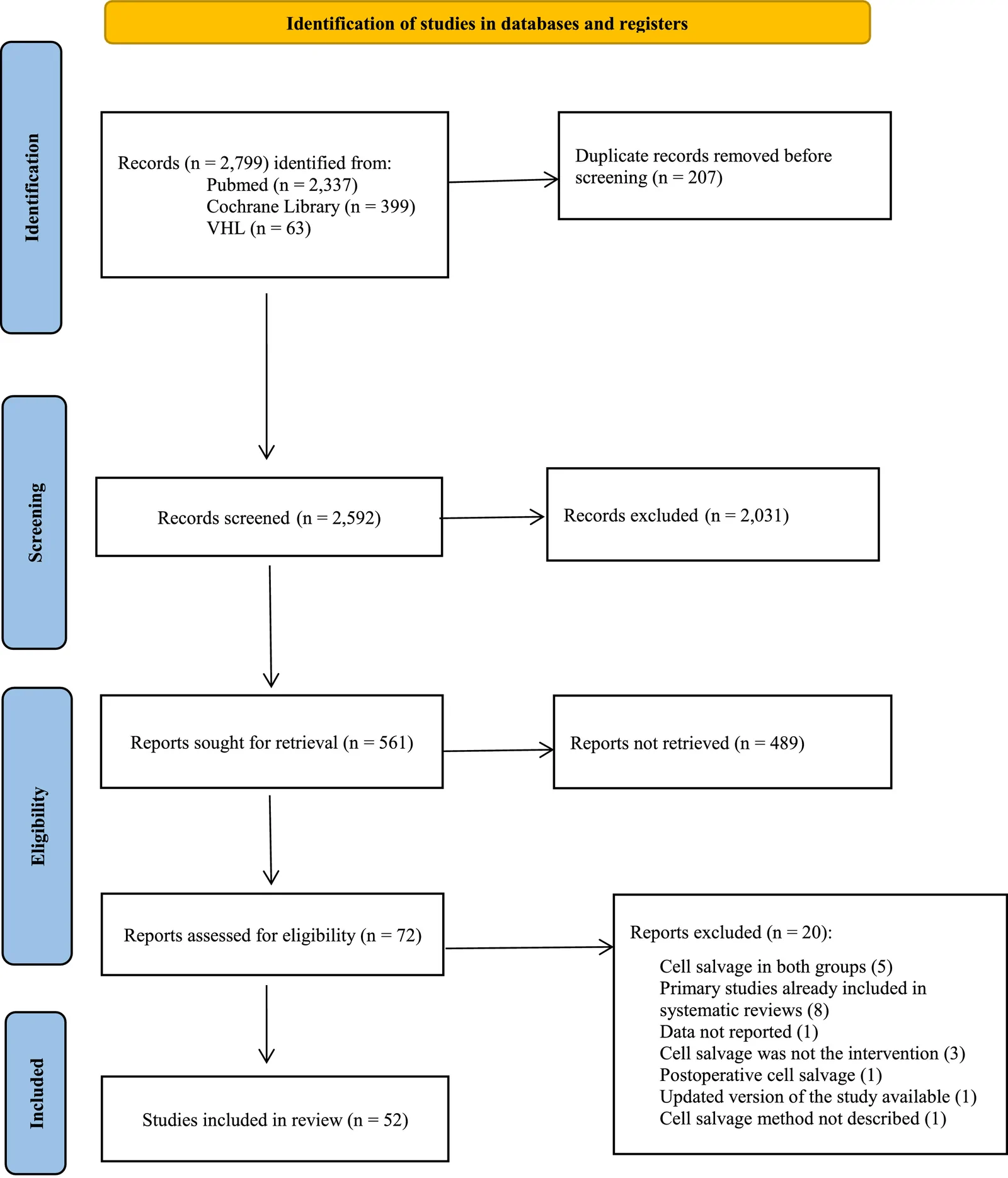
Process of including studies in the review.

A total of 2799 records were retrieved from electronic databases. After removing duplicates and screening titles and abstracts, 561 full-text articles were assessed for eligibility. Ultimately, 52 studies met the inclusion criteria and were included in the synthesis (Figure. 1). The excluded studies and the reasons for their exclusion can be found in Appendix 2. The included studies, published between 2009 and 2024, evaluated the use of intraoperative blood recovery across a wide range of surgical specialties, including cardiac [[2], [6], [7], [8], [9], [10], [11], [12], [13], [14]], orthopedic [[1], [2], [15], [16], [17], [18], [19], [20], [21], [22], [23]], oncologic [[2], [7], [24], [25], [26], [27]], urologic [[28], [29], [30], [31]], vascular [[2], [7]], obstetric [[2], [8], [32], [33], [34], [35], [36], [37], [38]], gastrointestinal [[39], [40], [41]], transplant [[41], [42], [43], [44], [45]], thoracic [46], pediatric [7], and trauma surgery [47].

VHL: Virtual Health Library

### Effectiveness of intraoperative blood recovery

Across most surgical contexts, intraoperative blood recovery demonstrated potential to reduce the need for allogeneic blood transfusion [2,[6], [7], [8], [9],^11^,^16^,^17^,^20^,^28^,^29^,[31], [32], [33], [34],[37], [38], [39], [40], [41], [42],^45^,[48], [49], [50], [51], [52], [53]]. The most consistent evidence of effectiveness was observed in orthopedic procedures, particularly spinal and joint replacement surgeries [2,7,16,17,20,[49], [50], [51]], obstetric interventions at high risk of hemorrhage [8,[32], [33], [34],^37^,^38^], gastrointestinal resections [39,40,53], and pediatric cardiac surgeries [7]. These settings demonstrated reductions in transfusion volume, transfusion rates, or both, particularly in studies using combined multimodal blood management strategies, such as acute normovolemic hemodilution or the use of tranexamic acid.

In cardiac surgery, the results were heterogeneous. While some studies reported a moderate reduction in exposure to allogeneic blood products, especially in off-pump coronary artery bypass grafting (OPCABG) [6], others reported no significant differences, especially in complex procedures involving cardiopulmonary bypass [2]. Similarly, in vascular [2] and thoracic surgeries [46], studies were inconclusive regarding the effectiveness of cell salvage, likely due to variability in surgical complexity and baseline blood loss.

Wang et al. [6] reported a 37% reduction in allogeneic transfusions and a 40% decrease in red blood cell use in cardiac surgeries. Similarly, Meybohm et al. [7] observed a 29% overall reduction in exposure to allogeneic blood products across surgical procedures, with a 45% decrease specifically in vascular surgeries.

In complex cardiovascular surgeries, Amanvermez et al. [9] found lower transfusion volumes (200 mL vs. 600 mL in controls). In orthopedics, Wong et al. [50] observed mean reinfusion volumes of 168.6 ± 89.3 mL (intraoperative) and 272.2 ± 104.2 mL (postoperative), while Herd et al. [51] reported 0.4 units per patient in the intervention group versus 3.5 in controls. In oncology surgery, studies reported a mean recovery of 1.21 red blood cell units per procedure. In obstetrics, a reduced need for allogeneic red blood cells and plasma was reported. Reinfused autologous blood volumes ranged from 300 mL [33] to 508.92 mL [38], and intraoperative cell salvage was associated with higher postpartum hemoglobin levels.

The use of cell salvage in oncologic surgery remains controversial particularly due to concerns about the reinfusion of malignant cells. Despite recent advances in leukocyte depletion filters, the results have been mixed. Some studies have demonstrated reduced transfusion needs without compromising oncological outcomes [26,27], while others have raised concerns about potential tumor recurrence in selected cancer types [25].

In urologic surgeries, cell salvage appeared effective in reducing transfusion needs in prostatectomies and nephrectomies, particularly in cases involving moderate to high blood loss [28,31,54]. However, the benefit was less clear in cystectomy procedures, where findings were inconsistent across studies [30].

### Safety and clinical outcomes

The safety profile of cell salvage was generally favorable across most surgical domains. Adverse events directly attributed to the technique were rare. Some studies reported reductions in postoperative infection [2,7,8,26], reoperation due to bleeding [26,27,29], or length of hospital stay [32,34,37,53], though these findings were not consistent across all populations or surgical types. In pediatric and oncologic contexts, specific concerns persisted, such as potential immunologic complications or recurrence risk, although no robust evidence has confirmed these risks [7,25].

In cardiac surgery, Luque-Oliveros et al. [11] reported a lower incidence of postoperative fever in the intraoperative cell salvage group (15%) compared to controls (25%), suggesting a potential benefit in reducing the inflammatory response.

Regarding safety, only the studies by Nunes et al. [55] and Wang et al. [32] reported the absence of complications related to cell salvage use, such as embolism and coagulopathy; other studies did not assess or report on these parameters.

Mortality rates also did not differ significantly in cardiac surgery [2], spine surgery [7], cystectomy [30], gastrointestinal surgery [53], vascular surgery [7], transplantation after one year [41], and trauma surgery [47]. Overall, intraoperative cell salvage appears safe, but does not significantly influence mortality based on current data.

### Costs

Sixteen studies evaluated the cost of intraoperative cell salvage compared to allogeneic transfusion, with mixed results: eight reported lower costs associated with intraoperative cell salvage [9,20,28,31,37,39,43,54], three demonstrated significantly higher costs [6,18,56], and five yielded inconclusive or neutral results [7,35,47,48,57].

Higher costs were observed primarily in studies conducted in Turkey and China involving orthopedic and cardiac surgeries. These increases were attributed to consumables (e.g., collection bags, filters), the need for additional personnel to operate the device, and extended intraoperative setup requirements. Wang et al. [32] reported that, compared with standard allogeneic transfusion, intraoperative cell salvage incurred consistently higher costs across all subgroups, with mean costs ranging from US$ 281.7 to US$ 439.2, depending on the extent of resource utilization. Similarly, Başaran et al. [18] and Bilgil et al. [56] found significantly higher perioperative transfusion costs in intraoperative cell salvage groups, largely due to resource-intensive implementation.

In contrast, eight studies conducted in countries including Brazil, Saudi Arabia, Australia, and the USA, identified cost savings from intraoperative cell salvage. These were largely due to reductions in allogeneic transfusion requirements, often by 1.4 to 1.5 units per patient. Amanvermez et al. [9] and Rajendran et al. [39] reported statistically significant cost reductions from intraoperative cell salvage, particularly in cardiac and gastrointestinal surgeries.

Five studies, mostly from the UK and South Africa, reported inconclusive results. These included randomized controlled trials and systematic reviews, which found either small, non-significant differences or variable outcomes, depending on the analytical approach (e.g., intention-to-treat vs. per protocol). Meybohm et al. [7] concluded that there is insufficient evidence to establish a consistent cost-effectiveness profile for intraoperative cell salvage.

## Discussion

This rapid review provides a summarized and contextualized overview of the current evidence on intraoperative blood recovery, to identify its potential for reducing the need for allogeneic transfusions across multiple surgical contexts. The findings corroborate previous literature suggesting that cell salvage can offer both clinical and systemic benefits, including improved patient safety and resource optimization. Nevertheless, the variation in effectiveness across surgical specialties and the mixed results regarding costs highlight the need for context-specific evaluations before broader implementation. In summary, the costs of intraoperative cell salvage vary according to the surgical context, healthcare infrastructure, and reimbursement systems. Although initial investment may be higher in some settings, reductions in allogeneic transfusion requirements may offset these costs in certain clinical contexts [9,39].

Recent evidence further supports the role of intraoperative blood recovery as a component of patient blood management (PBM) strategies, particularly in surgeries with high bleeding risks. In cardiac surgery, guidelines call for the integration of PBM practices, highlighting the role of cell salvage in minimizing transfusion-related complications [58]. Similarly, the French National Health Authority recommends the use of cell salvage techniques in surgeries with a high risk of transfusion, reinforcing the importance of intraoperative blood recovery in contemporary surgical care [59]. Globally, the 2025 World Health Organization guidelines advocate for the broader implementation of PBM to improve patient safety and promote the efficient use of healthcare resources, thereby reinforcing the importance of cell salvage in modern surgical care [60].

The safety profile of intraoperative blood recovery remains favorable, with studies reporting low incidences of adverse events directly attributable to the procedure. Multiple studies in both obstetric and general surgical settings report that cell salvage is safe, with no significant adverse effects directly linked to the procedure itself [34,38].

The quality of most systematic reviews was rated as low [7,31,48] or critically low [15,16,19,26,37,39] due to issues such as unregistered protocols and inadequate bias assessment. Randomized trials generally were of higher quality [13,34,35,38], despite some limitations in blinding and follow-up. Cohort [8,10,11,14,36,[43], [44], [45]] and case-control studies [30,32,33,42,53] were of moderate quality, with variability in confounder control and reporting. Economic evaluations were the weakest [6,9,18,28,54], often lacking detail, sensitivity analysis, or model transparency, and of limited usefulness for decision-making.

This review did not aim to capture all available evidence on the effectiveness of cell salvage. Its scope was limited by methodological constraints typical of rapid reviews, including the restriction to three databases, which may have led to omission of relevant studies, particularly those published in non-indexed languages or within the grey literature.

Evidence synthesis was further complicated by the heterogeneity of the included studies, both in methodological approaches and outcome reporting. Many studies lacked complete or consistent data, and more robust comparative analyses were limited by insufficient detail in statistical reporting. Additionally, poor reporting quality, lack of statistically significant outcomes, and inconsistent criteria for assessing effectiveness and safety hindered comparability and may have affected the overall validity of the findings.

As an initial synthesis with a broad scope across multiple surgical specialties, this review provides a comprehensive overview of the current evidence on intraoperative blood recovery. However, further research with greater methodological rigor is warranted, including systematic reviews with expanded search strategies and well-designed primary studies, such as prospective cohort or case-control studies, to better assess setting-specific efficacy and cost-effectiveness.

For clinicians and policymakers, this review indicates that cell salvage is a clinically safe and potentially cost-effective intervention in selected surgical contexts. Implementation should be guided by local transfusion needs, healthcare infrastructure, and available resources. A targeted approach is recommended, focused on high-transfusion-risk procedures and ensuring appropriate clinical training and equipment maintenance.

Future research should prioritize long-term clinical outcomes, patient-reported measures, and real-world cost-effectiveness. Equity-focused studies are also needed to assess the potential of cell salvage to improve access to transfusion support in underserved populations.

## Data availability

The data that support the findings of this study are available from the corresponding author upon reasonable request.

## Conflicts of interest

The authors declare no conflict of interest.

## Figures and Tables

**Table:** 

**Database**	**Search strategy**	**Results**
PubMed	((("Surgical procedures, operative"[MeSH Terms] OR "Surgical Procedure*" OR "General Surgery"[Mesh] OR "surgery" [Subheading] OR Surgery[Title/Abstract])) AND (("Blood Transfusion, Autologous"[Mesh] OR "Blood Transfusion"[Mesh] OR "Blood Transfusion*" OR Transfusion* OR "Blood Preservation"[Mesh] OR "Blood Preservation*" OR "Autologous salvaged blood" OR Autotransfusion))) AND (("Operative Blood Salvage"[Mesh] OR "Intraoperative Blood Salvage" OR "Cell Saver" OR "Cell Savers" OR " Cell salvage" OR "Cell saving" OR "Blood Cell Salvage" OR "Intraoperative cell salvage" OR "Patient blood management" OR "Blood Cell Salvage" OR "Salvaged blood" OR "Blood Salvage"))	2337
Cochrane Library	#1MeSH descriptor: [Surgical Procedures, Operative] explode all trees #2("Surgical Procedure" OR "Surgical Procedures"):ti,ab,kw #3MeSH descriptor: [General Surgery] explode all trees #4(Surgery):ti,ab,kw #5MeSH descriptor: [] explode all trees and with qualifier(s): [surgery - SU] #6MeSH descriptor: [Blood Transfusion, Autologous] explode all trees #7MeSH descriptor: [Blood Transfusion] explode all trees #8("Blood Transfusion" OR Transfusion* OR Autotransfusion):ti,ab,kw #9MeSH descriptor: [Blood Preservation] explode all trees #10("Blood Preservation" OR "Autologous salvaged blood"):ti,ab,kw #11MeSH descriptor: [Operative Blood Salvage] explode all trees #12("Intraoperative Blood Salvage" OR "Cell Saver" OR "Cell Savers" OR " Cell salvage" OR "Cell saving" OR "Blood Cell Salvage" OR "Intraoperative cell salvage" OR "Patient blood management" OR "Blood Cell Salvage" OR "Salvaged blood" OR “Blood Salvage”):ti,ab,kw #13{OR #1-#5} #14{OR #6-#10} #15#11 OR #12 #16#13 AND #14 AND #15	399
VHL	"cell saver" OR "cell savers" OR "cell salvage" OR "blood cell salvage" OR "blood salvage" OR "intraoperative cell salvage" AND (db:("LILACS" OR "IBECS" OR "BDENF" OR "BIGG" OR "CUMED"))	63
**Total**		**2799**

**Table:** 

Author/ Year	Title	Reason for exclusion
Adam et al. 2020 [61]	Impact of Intraoperative Cell Salvage on Blood Coagulation Factor Concentrations in Patients Undergoing Cardiac Surgery	The cell saver was used in the intervention and control groups
Alamanda et al. 2018 [62]	Blood Loss Trends and Financial Implications in Adolescent Idiopathic Scoliosis	The cell saver was used in the intervention and control groups
Buget et al. 2016 [63]	Two-year experience with cell salvage in total hip arthroplasty	The method was not described
Carless et al. 2004 [64]	Autologous transfusion techniques: a systematic review of their efficacy	The data did not separate how the technique was used during surgery
Carless et al. 2010 [65]	Cell salvage for minimizing perioperative allogeneic blood transfusion	Used the Loyd 2023 study (update)
Henderson et al. 2019 [66]	Impact of intraoperative high-volume autologous blood collection on allogeneic transfusion during and after cardiac surgery: a propensity score matched analysis	The comparator was not allogeneic transfusion
Hong et al. 2016 [67]	Comparison between autologous blood transfusion drainage and closed-suction drainage/no drainage in total knee arthroplasty: a meta-analysis	The comparator was not allogeneic transfusion
Liang et al. 2015 [68]	Does intraoperative cell salvage system effectively decrease the need for allogeneic transfusions in scoliotic patients undergoing posterior spinal fusion? A prospective randomized study	Study included in the Liu 2017 review
Lyon et al. 2015 [69]	Short-term Outcomes of Intraoperative Cell Saver Transfusion During Open Partial Nephrectomy	Study included in the Kinnear 2017 review
Miao et al. 2014 [70]	The efficacy and cost-effectiveness of cell saver use in instrumented posterior correction and fusion surgery for scoliosis in school-aged children and adolescents	Study included in the Liu 2017 review
Miao et al. 2024 [71]	Intraoperative cell salvage reduces postoperative allogeneic blood transfusion and shortens off-bed time in simultaneous bilateral total hip arthroplasty: a single-center retrospective study	The comparator group received allogeneic transfusion not intraoperatively, but postoperatively
Pabon-Carrasco et al. 2024 [72]	The effectiveness of cell salvage in extracorporeal circulation surgeries in relation to use of health resources after use: A systematic review and meta-analysis	The comparator was not allogeneic transfusion
Pan et al. 2016 [73]	The efficacy and safety of autologous blood transfusion drainage in patients undergoing total knee arthroplasty: a meta-analysis of 16 randomized controlled trials	The comparator was not allogeneic transfusion
Pinto et al. 2021 [74]	Impact of intraoperative blood salvage and autologous transfusion during liver transplantation for hepatocellular carcinoma	Study included in the Rajendran 2023 review
Sullivan & Ralph 2019 [75]	Obstetric intra-operative cell salvage: a review of an established cell salvage service with 1170 re-infused cases	The cell saver was used in the intervention and control groups
Vermeijden et al. 2015 [76]	Effects of cell-saving devices and filters on transfusion in cardiac surgery: a multicenter randomized study	Study included in the Meybohm 2016 review
Xie et al. 2015 [77]	The efficacy, safety and cost-effectiveness of intra-operative cell salvage in high-bleeding-risk cardiac surgery with cardiopulmonary bypass: a prospective randomized and controlled trial	Study included in the Meybohm 2016 review
Yang et al. 2017 [78]	Evaluation of acute normovolemic hemodilution in patients undergoing intracranial meningioma resection: a quasi-experimental trial	The cell saver was used in the intervention and control groups
Zacharias et al. 2018 [79]	Intraoperative cell salvage with autologous transfusion in elective right or repeat hepatectomy: a propensity-score-matched case-control analysis	Study included in the Rajendran 2023 review
Zhou et al. 2015 [80]	Autologous platelet-rich plasma reduces transfusions during ascending aortic arch repair: a prospective, randomized, controlled trial	The cell saver was used in the intervention and control groups

## Appendix 1 Search strategies

Access date: 02 October 2024.
